# From Genes to Greens: How Diet, Climate and Phylogeny Shape Freshwater Turtle Microbiotas

**DOI:** 10.1111/1462-2920.70396

**Published:** 2026-08-06

**Authors:** T. Franciscus Scheelings, Thi Thu Hao Van, Anthony Santoro, Louise M. Streeting, Christopher T. Ormond, James U. Van Dyke, Lee F. Skerratt, Robert J. Moore

**Affiliations:** ^1^ Melbourne Veterinary School, Faculty of Science The University of Melbourne Werribee Victoria Australia; ^2^ School of Science RMIT University Bundoora Victoria Australia; ^3^ Centre for Sustainable Aquatic Ecosystems Harry Butler Institute, Murdoch University Murdoch Western Australia Australia; ^4^ School of Environmental and Rural Science University of New England Armidale New South Wales Australia; ^5^ Department of Climate Change, Energy, the Environment and Water New South Wales Government Coffs Harbour New South Wales Australia; ^6^ Centre for Freshwater Ecosystems, Department of Ecological, Plant and Animal Sciences School of Agriculture, Biomedicine and Environment, La Trobe University West Wodonga Victoria Australia

**Keywords:** climate, diet, freshwater turtles, microbiota, phylogeny

## Abstract

Reptile microbiotas remain poorly understood despite their importance for host ecology and evolution. This study investigated how diet, climate and evolutionary history shape the gut microbiota of 234 Australian freshwater turtles spanning 10 species, four climatic zones and contrasting trophic strategies. It was found that turtles' microbiotas were dominated by Pseudomonadota, Actinobacteriota and Bacteroidota. Microbial richness and evenness varied significantly among species, diets and climates. Carnivorous turtles exhibited greater bacterial diversity than omnivores, while individuals from oceanic and humid subtropical zones had higher diversity than those from semi‐arid and Mediterranean regions. Canonical Correspondence Analysis confirmed diet and climate as significant predictors of community composition, together explaining 2.76% of total variation. Omnivorous turtles were enriched in Bacteroidota, Myxococcota, Cyanobacteriota and Bacillota, whereas climatic effects drove distinct phylum‐level signatures across habitats. Host phylogeny showed a weak but significant signal of phylosymbiosis, indicating an evolutionary imprint on microbial structure. Collectively, these findings reveal that turtle microbiotas are shaped by a complex interplay between ecological and evolutionary forces, underscoring the importance of integrating microbial data into conservation strategies for Australia's increasingly threatened freshwater turtles.

## Graphical abstract

Gut microbiotas of 234 Australian freshwater turtles vary with diet, climate and evolutionary history. Carnivorous turtles hosted richer, distinct microbial communities compared with omnivores, while climate zones shaped phylum‐level patterns. Ecological factors dominated over phylogeny, highlighting the importance of integrating microbiome and dietary data into turtle conservation.

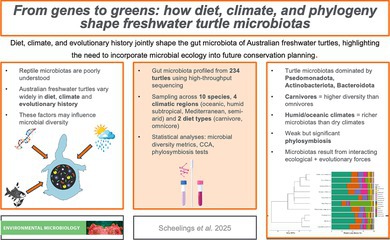

## 1 Background

Most metazoans host a vast and dynamic community of microorganisms, collectively known as the microbiota, that contributes profoundly to the health, ecology and evolution of its host (Moran et al. 2019). The concept of the holobiont: the host and its microbiota functioning as a single ecological and evolutionary unit, has transformed our understanding of animal biology (Theis et al. 2016). The combined genetic material of this partnership, referred to as the hologenome, plays a fundamental role in shaping physiological traits, immune responses and adaptive capacities (Theis et al. 2016).

The gastrointestinal tract is a critical site of microbial colonisation in animals, where complex microbial consortia facilitate digestion (Hanning and Diaz‐Sanchez 2015), nutrient assimilation (Rudoy et al. 2023) and immune regulation (Kim 2018). Compared to other vertebrate groups, studies of reptile gut microbiota are relatively scarce (Colston and Jackson 2016; Hoffbeck et al. 2023). Yet, it is increasingly evident that these microbial communities play vital roles in host development, metabolism and resilience to environmental change. Despite growing recognition of their importance, microbiota research in Australian freshwater turtles is particularly depauperate and the ecological and evolutionary drivers of microbiota composition are virtually unexplored (Scheelings et al. 2024, 2025).

In chelonians, as in other vertebrates, the microbiota is increasingly recognised as a heritable and evolutionarily significant trait (Scheelings et al. 2020). Evidence from diverse animal systems indicates that microbial community composition is not random but influenced by host phylogeny, suggesting that hosts and their microbiota have co‐evolved over millions of years (Ley et al. 2008; Ochman et al. 2010; Hird et al. 2015; Lilli et al. 2023). This reciprocal relationship between microbial symbionts and host physiology has likely guided the diversification of dietary niches and ecological adaptations across evolutionary time (Kujawska et al. 2025). Understanding these interactions requires integrating microbiological, ecological and phylogenetic approaches to uncover how evolutionary forces have acted upon both host and microbial genomes.

With the exception of the pig‐nosed turtle ( *Carettochelys insculpta* ) all Australian freshwater turtles represent a single clade, the Pleurodira, making them an ideal group in which to explore these questions. They are an ancient lineage of reptiles that exhibit substantial ecological diversity, including variation in habitat use, life‐history strategies and trophic ecology. This diversity provides a valuable framework for investigating the ecological and evolutionary factors shaping host–microbiome interactions. Their distribution spans a wide range of climatic zones, from tropical northern rivers to temperate southern wetlands (Cogger 2014), providing natural variation in environmental conditions that may shape microbial assemblages. Unlike many other vertebrate systems, this group allows investigation of microbial diversity across closely related species within a contained evolutionary framework, reducing confounding effects of deep evolutionary divergence.

Multiple factors influence microbial diversity within and among species, including diet (Bloodgood et al. 2020), habitat type (Parks et al. 2024), geography (Scheelings et al. 2024; Madison et al. 2022), temperature (Sepulveda and Moeller 2020) and captivity status (Dallas and Warne 2023; Jenkins et al. 2024). Yet, disentangling these ecological influences from the underlying effects of host evolutionary history remains a critical challenge. Determining whether closely related turtle species harbour more similar microbiotas than distant relatives can reveal the extent to which microbial assemblages reflect phylogenetic inheritance versus environmental adaptation.

Understanding how microbial communities have co‐evolved with reptiles is not only fundamental from an evolutionary perspective but also has important implications for conservation (Jenkins et al. 2024; Jiang et al. 2025; Hoffbeck 2025). This is especially pertinent for chelonians, which are among the most imperilled vertebrates on the planet (Stanford et al. 2020). In Australia, nearly half of the freshwater turtle species are listed as threatened (Van Dyke et al. 2018), highlighting the urgent need for a holistic approach to conservation to arrest these dramatic population declines. Given the undeniable links between gut microbial composition and host physiology, characterising the microbiota and its determinants may offer insights into the resilience and adaptability of these species under environmental stress.

In this study, the microbiota of Australian freshwater turtles across multiple species, diets and climatic regions was examined. By integrating microbiota profiling with host phylogenetic analyses, the aim was to (i) characterise the composition and diversity of turtle‐associated microbial communities, (ii) determine the relative influence of diet, environment and host evolutionary relationships on microbiota structure and (iii) explore how these microbial partnerships may have shaped the ecological and evolutionary trajectories of freshwater turtles in Australia.

## 2 Methods

### 2.1 Study Populations

Turtles were trapped and sampled under permit 10010480 from the Department of Environment, Land, Water and Planning (VIC) and permit number RP1497 from the Victorian Fisheries Authority (VIC), permit SL102774 from the Department of Climate Change, Energy, the Environment and Water (NSW), permits TFA 2324‐0037 and FO25000094‐9 from the Department of Biodiversity, Conservation and Attractions (WA), Fisheries Exemption 251252824 (WA), permit number 266061 from the Department of Agriculture and Fisheries (QLD) and permit number WA0048559 from the Department of Environment and Science (QLD). All turtles were released alive at their point of capture immediately after sampling had been completed.

Turtles were captured from multiple sites in four Australian states (Table 1). The species captured for this investigation included at least one representative of 6 of the 7 extant genera of freshwater turtles in Australia. The only genera that we were unable to sample were *Pseudemydura*, which contains the monotypic species the western swamp turtle ( *P. umbrina* ). We assigned dietary preferences based on peer‐reviewed natural history data and climate categories were determined using the Köppen classification system from the Australian Bureau of Meteorology (Meterology Bo, n.d.).

**TABLE 1:** Summary of Australian freshwater turtle species sampled for microbiota analysis, including the number of individuals per species, capture sites, dietary categories and the climate zones of each sampling location.

Species	Total	Location	Diet	Climate
*Chelodina* (Long‐necked turtles)				
Broad‐shell turtle ( *Chelodina expansa* )	11	Moloney Wetland (VIC, *n* = 8) Mary River (QLD, *n* = 3)	Carnivore (Chessman 1983)	Humid subtropical Humid subtropical
Eastern long‐neck turtle ( *Chelodina longicollis* )	78	Baranduda (VIC, *n* = 20) Barnawartha (VIC, *n* = 18) Darebin Wetlands (VIC, *n* = 40)	Carnivore (Kennett et al. 2009)	Humid subtropical Humid subtropical Oceanic
Oblong turtle ( *Chelodina oblonga* )	20	Lake Claremont (WA, *n* = 10) Baigup Wetlands (WA, *n* = 10)	Carnivore (Woldering 2001)	Hot‐summer mediterranean
*Elseya* (Snapping turtles)				
White‐throated snapping turtle ( *Elseya albagula* )	21	Fitzroy River (QLD, *n* = 7) Mary River (QLD, *n* = 14)	Omnivore (Micheli‐Campbell et al. 2017)	Hot semi‐arid Humid subtropical
*Elusor*				
Mary River turtle ( *Elusor macrurus* )	8	Fitzroy River (QLD, *n* = 8)	Omnivore (Cann and Legler 1994)	Humid subtropical
*Emydura*				
Macquarie River turtle ( *Emydura macquarii* )	48	Bellinger River (NSW, *n* = 7) Maloney Wetland (VIC, *n* = 9) Baranduda (VIC, *n* = 6) Fitzroy River (QLD, *n* = 10) Mary River (QLD, *n* = 16)	Omnivore (Spencer et al. 1998)	Humid subtropical Humid subtropical Humid subtropical Hot semi‐arid Humid subtropical
*Rheodytes*				
Fitzroy River turtle ( *Rheodytes leukops* )	8	Fitzroy River (QLD, *n* = 8)	Omnivore (Tucker et al. 2001)	Hot semi‐arid
*Myuchelys*				
Western saw‐shelled turtle ( *Myuchelys bellii* )	17	Kentucky Reservoir (NSW, *n* = 17)	Omnivore (Fielder et al. 2015)	Oceanic
Bellinger River turtle ( *Myuchelys georgesi* )	32	Bellinger River (NSW, *n* = 32)	Omnivore (Cann et al. 2015)	Humid subtropical
Saw‐shelled turtle ( *Myuchelys latisternum* )	14	Mary River (QLD, *n* = 14)	Omnivore (Tucker et al. 2012)	Humid subtropical

Abbreviations: NSW = New South Wales; QLD = Queensland; VIC = Victoria; WA = Western Australia.

### 2.2 Sample Collection

At all sites, turtles were captured using a variety of methods including baited and unbaited fyke nets, baited cathedral nets, modified funnel traps or by hand capture while snorkelling. The capture method used at each location was selected based on the behavioural characteristics of the target species and the experience of the field team. Following capture, each turtle was placed in dorsal recumbency and a sterile cotton swab (Swab Stick Sterile 15 cm 18165‐MC) was inserted into the cloaca and twirled so that it contacted the cloacal mucosa. The swab was then retracted and the tip cut using flame‐sterilised wire cutters and stored in 1 mL of ZymoBIOMICS DNA/RNA Shield (Integrated Sciences) in a sterile Eppendorf tube. At each field site, 2 swabs were opened and then placed into a sterile Eppendorf tube as negative controls. The Eppendorf tubes were then frozen and stored at −80°C until DNA extraction could occur. All turtles were released alive at their point of capture following sample collection.

### 2.3 DNA Extraction

DNA was extracted using the ZymoBIOMICS DNA Miniprep Kit (Integrated Sciences) according to the manufacturer's instructions. This method was selected because validation work by the manufacturer had demonstrated excellent quantitative recovery of microbial DNAs. Following extraction, DNA was stored at −80°C until amplicon sequencing could take place. Negative extraction controls (extraction reagents without biological material) were processed and sequenced alongside study samples to monitor contamination and assess extraction and sequencing performance.

### 2.4 16S rRNA Gene Amplicon Sequencing

The V3‐V4 region of 16S rRNA genes was PCR amplified with forward primer 5′ ACTCCTACGGGAGGCAGCAG 3′ and reverse primer 5′ GGACTACHVGGGTWTCTAAT 3′ using Q5 high fidelity polymerase (New England Biolabs) with a dual barcoding strategy (Fadrosh et al. 2014). The PCR cycling parameters were 98°C for 1 min, 35 cycles of 98°C for 10 s, 49°C for 30 s and 72°C for 30 s, followed by a 10‐min extension at 72°C. Sequencing was performed on an Illumina MiSeq system (2 × 300 bp).

### 2.5 Data Processing

Sequence data was analysed using Quantitative Insights into Microbial Ecology 2 (QIIME2) version 220.6 (Bolyen et al. 2019), using the Divisive Amplicon Denoising Algorithm (DADA2) plugin for quality filtering, denoising, chimaera detection and amplicon sequence variant (ASV) calling (Callahan et al. 2016). Trimming was done in DADA2 using the default parameters and ASVs with fewer than 10 sequence reads across the entire data set were removed. ASVs were taxonomically classified using the SILVA database (v138.1) (Quast et al. 2013) and an ASV abundance table with taxonomic assignments was produced for further analysis.

### 2.6 Statistical Analyses

ASV abundance data was analysed in R, utilising the packages ‘phyloseq’ (McMurdie and Holmes 2013), ‘vegan’ (Oksanen et al. 2025) and ‘microeco’ (Liu et al. 2021). Alpha diversity was explored using Observed ASVs, Shannon index and Inverse Simpson estimates. Alpha diversity was tested for normality using the Shapiro–Wilks test and when data was non‐normally distributed comparisons between locations were first made using a Kruskal–Wallis test and then pairwise comparisons were made using the Wilcoxon rank sum test. We tested alpha diversity metrics for species, diet and climate. Beta diversity was initially investigated using Principal Co‐ordinate Analysis (PCoA) on weighted and unweighted UniFrac distances. We used the *adonis2* function from the R package ‘vegan’ to perform PERMANOVAs and then pairwise comparisons were made between all combinations of species (Oksanen et al. 2025). We further examined beta diversity using Canonical Correspondence Analysis (CCA), incorporating diet and climate into the model to assess their influence on bacterial community composition. To identify which bacterial phyla were driving these differences, we performed differential abundance testing using the package ‘DESeq2’ (Love et al. 2014). For abundance testing read counts were transformed to proportions per sample (i.e., total sum normalisation (TSN)) prior to calculating the distances and dissimilarities (McKnight et al. 2018).

To investigate the relationships between host phylogeny and microbiota composition we obtained a host phylogenetic tree using the online program TimeTree 5 (Kumar et al. 2022). We then incorporated this into our phyloseq data and ran Mantel and PERMANOVA tests. To further evaluate which bacterial genera were associated with phylosymbiosis we performed a distance‐based redundancy analysis (dbRDA) using the ‘vegan’ package. A Bray–Curtis dissimilarity matrix was calculated from the TSN ASV abundance data using the vegdist function. The host phylogenetic tree was used as the explanatory variable (constraint) in the dbRDA model via the capscale function. This analysis partitions the microbial community variation into components explained by the host phylogeny. The significance of the model was then tested using an ANOVA test. The results of the dbRDA were illustrated using a PCoA plot to visualise samples and the top 20 most abundant genera contributing to the primary constrained axis (CAP1). To highlight the importance of individual genera to the host‐associated variation, a bar plot was created. The CAP1 scores for all ASVs belonging to the top 20 genera were averaged and presented as mean contribution scores along the CAP1 axis. For all pairwise tests, *p*‐values were adjusted for multiple comparisons with Bonferroni correction. For all statistical analyses significance was accepted if *p* < 0.05.

## 3 Results

**FIGURE 1:**
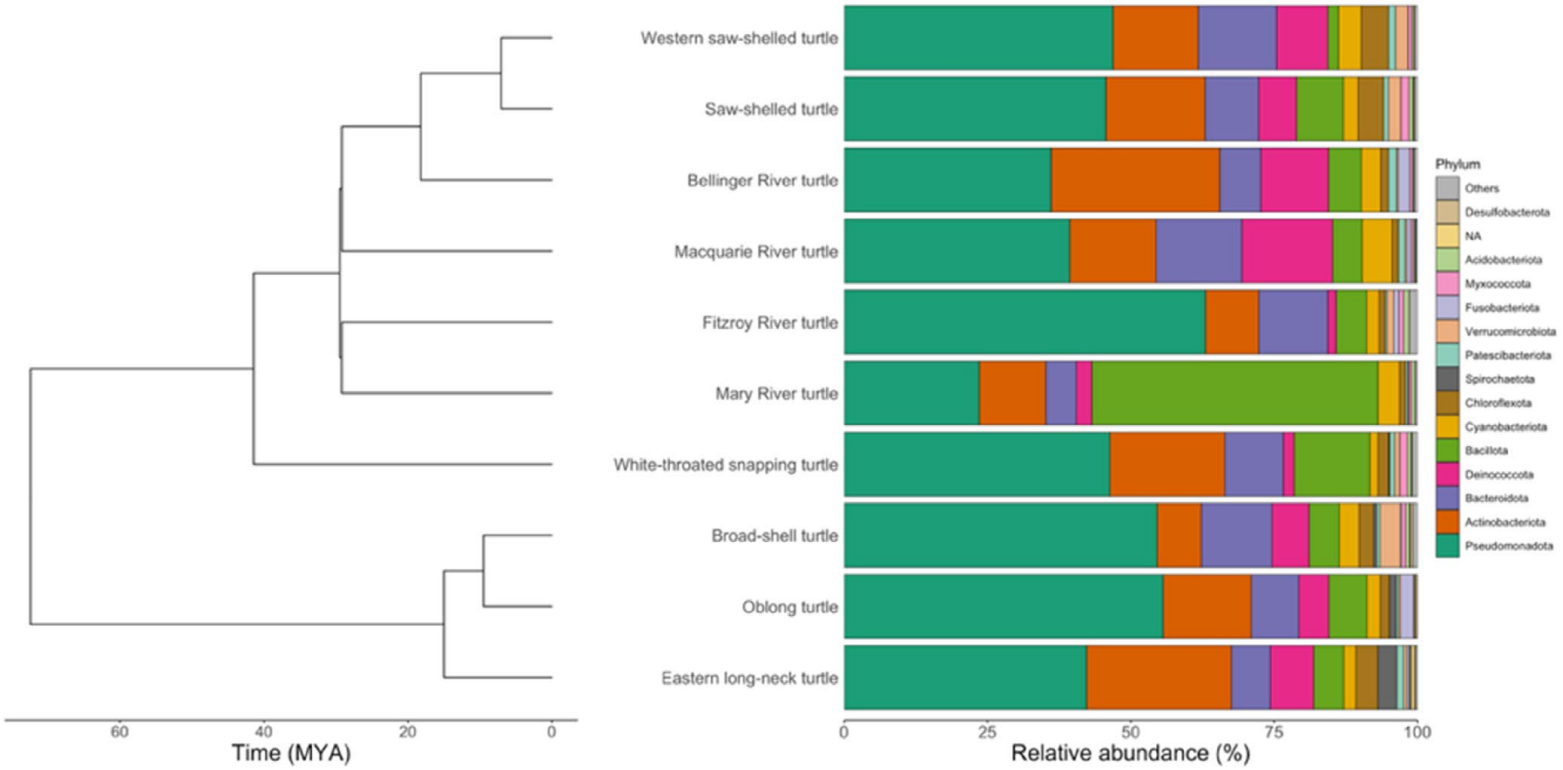
Microbiota composition with host phylogeny of Australian freshwater turtles. The stacked bar plots represent the relative abundance of bacterial phyla within each turtle species, while the accompanying phylogenetic tree depicts host evolutionary relationships. The alignment of microbiota profiles with host phylogeny illustrates patterns of phylosymbiosis, where closely related turtle species exhibit more similar microbial community structures.

In total, 234 turtles were captured and sampled (Table 1). A total of 5,812,296 sequences were generated after quality checking and removal of chimaeras, giving an average of 22,616 sequences per sample. The taxonomic breakdown of sequence data yielded 38 bacterial phyla, 95 classes, 251 orders, 442 families, 415 genera, 1058 species and 2971 ASVs. In our dataset the most abundant phylum in freshwater turtles was Pseudomonadota (43.5%), followed by Actinobacteriota (19.9%), Bacteroidota (9.7%), Deinococcota (8.7%) and Bacillota (7.4%) (Figure 1).

**FIGURE 2:**
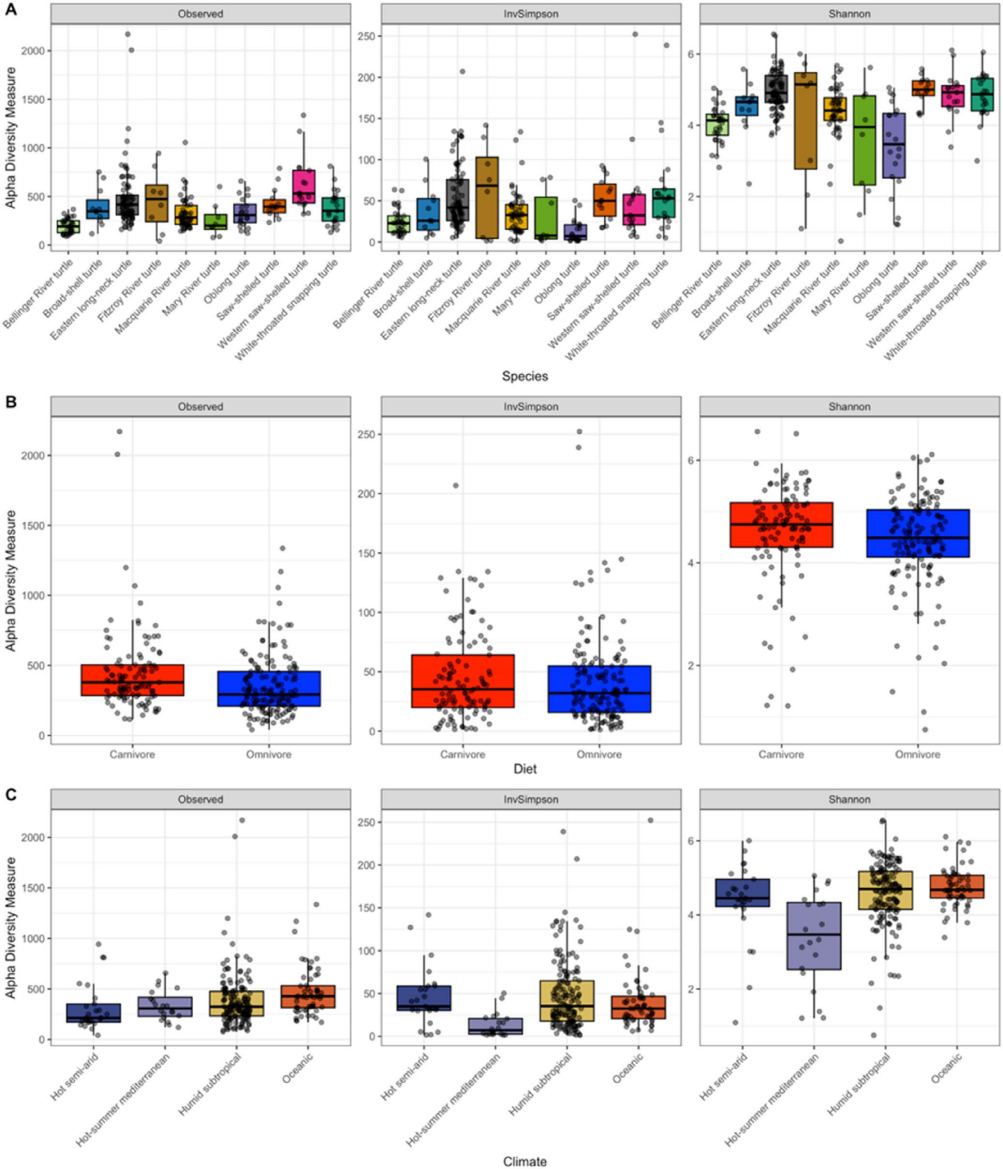
Microbiota alpha diversity analysis of cloacal samples of wild freshwater turtles from Australia. Boxplots illustrate diversity using Observed, Inverse Simpson and Shannon diversity indices in bacterial communities between species (A), between dietary preferences (B) and between climate (C).

**FIGURE 3:**
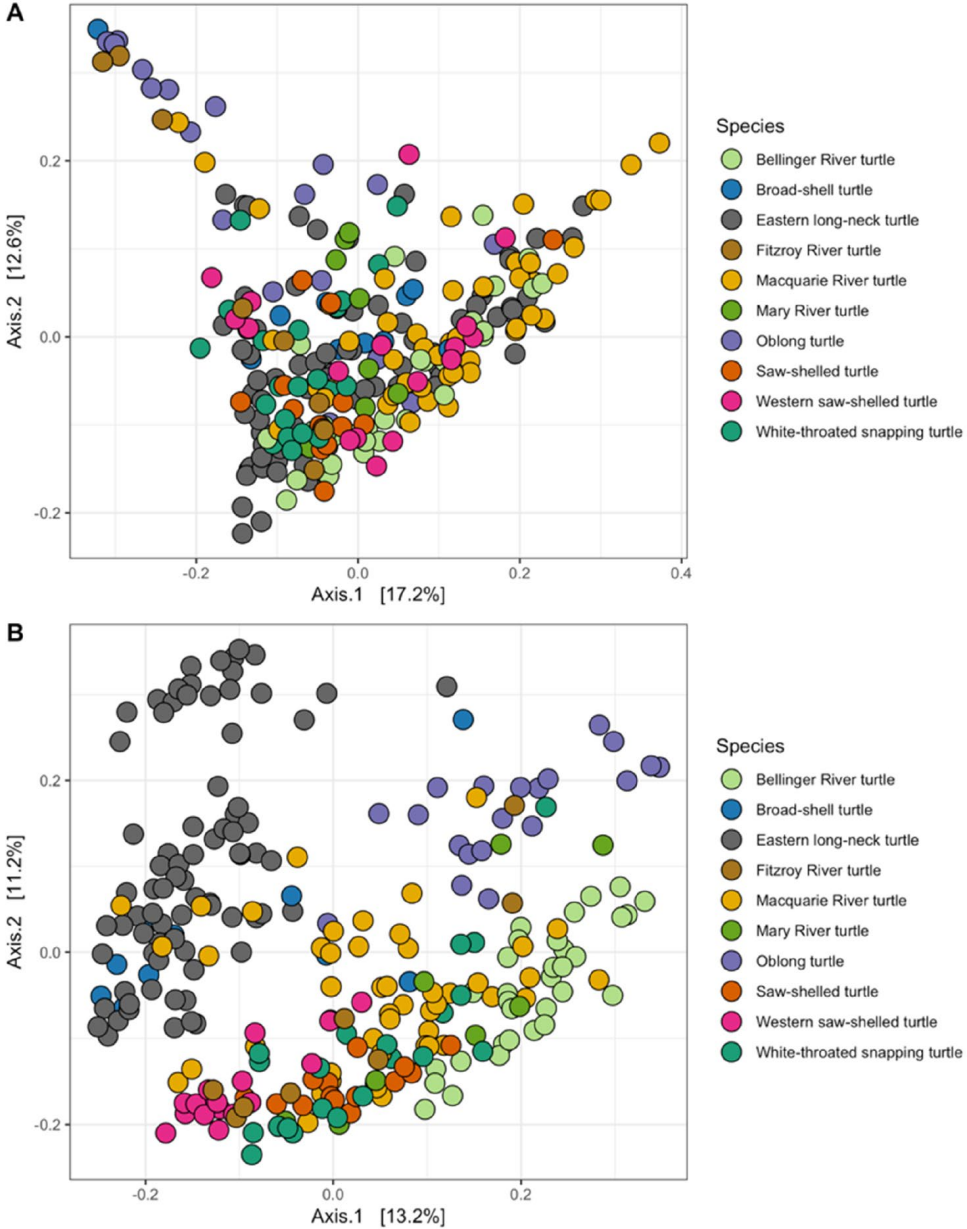
Microbiota beta diversity analysis of cloacal samples in wild Australian freshwater turtles. Diversity was measured using Principal Coordinate Analysis (PCoA) plots based on weighted UniFrac (A) and unweighted UniFrac (B) distances at a genus level and showed that significant differences existed between species.

For turtle cloacal alpha diversity, Observed (*W* = 0.78, *p* < 0.01), Inverse Simpson (*W* = 0.83, *p* < 0.01) and Shannon (*W* = 0.91, *p* < 0.01) were all non‐normally distributed. Analysis of alpha diversity at a species level revealed that there were significant differences between species for Observed ASVs (*χ* ^2^ = 76.4, df = 9, *p* < 0.01), Inverse Simpson (*χ* ^2^ = 51.75, df = 9, *p* < 0.01) and for Shannon (*χ* ^2^ = 74.5, df = 9, *p* < 0.01) (Figure 3A). Pairwise comparisons between combinations of species identified that most detectable differences existed for Observed in Bellinger River turtles and other species with few differences noted between other combinations (Additional File 1, Tables S1–S3). For diet, we found that carnivores had a greater number of Observed ASVs (*χ* ^2^ = 12.64, df = 1, *p* = 0.02) and Shannon diversity (*χ* ^2^ = 6.0, df = 1, *p* = 0.01) in comparison to omnivores, but we did not detect any differences for Inverse Simpson (*χ* ^2^ = 0.66, df = 1, *p* = 0.42) (Figure 3B). Similarly, when we analysed climate data, we found that differences were apparent for Observed ASVs (*χ* ^2^ = 16.37, df = 3, *p* < 0.01), Inverse Simpson (*χ* ^2^ = 25.35, df = 3, *p* < 0.01) and Shannon (*χ* ^2^ = 20.66, df = 3, p < 0.01) (Figure 2C) when these metrics were analysed together. Pairwise comparisons between combinations of climates identified significant differences between multiple combinations for all indices with animals originating from oceanic climates consistently having higher alpha diversity metrics than most other climatic zones except for humid subtropical (Additional File 1, Tables S4–S6).

For beta diversity PERMANOVA of weighted UniFrac revealed a significant difference in the overall microbial community composition between species (df = 9, SST = 5.87, *R* ^2^ = 0.22, f.model = 8.03, *p* < 0.01) (Figure 3A). The results of beta dispersion testing indicated a significant difference in the dispersion of microbial communities across the species examined (df = 9, SST = 0.18, MS = 0.02, f.model = 3.19, *p* < 0.01). Similarly, PERMANOVA detected a significant difference in overall microbial community composition across the experimental groups for unweighted UniFrac (df = 9, SST = 12.77, *R* ^2^ = 0.27, f.model = 10.1, *p* < 0.01) (Figure 3B), suggesting that community structures differed between species. And like weighted UniFrac, we also had a significant difference when beta dispersion was tested (df = 9, SST = 0.33, MS = 0.04, f.model = 7.54, *p* < 0.01). Post hoc pairwise comparisons are shown for all combinations in Additional File 1 (Tables S7 and S8).

**FIGURE 4:**
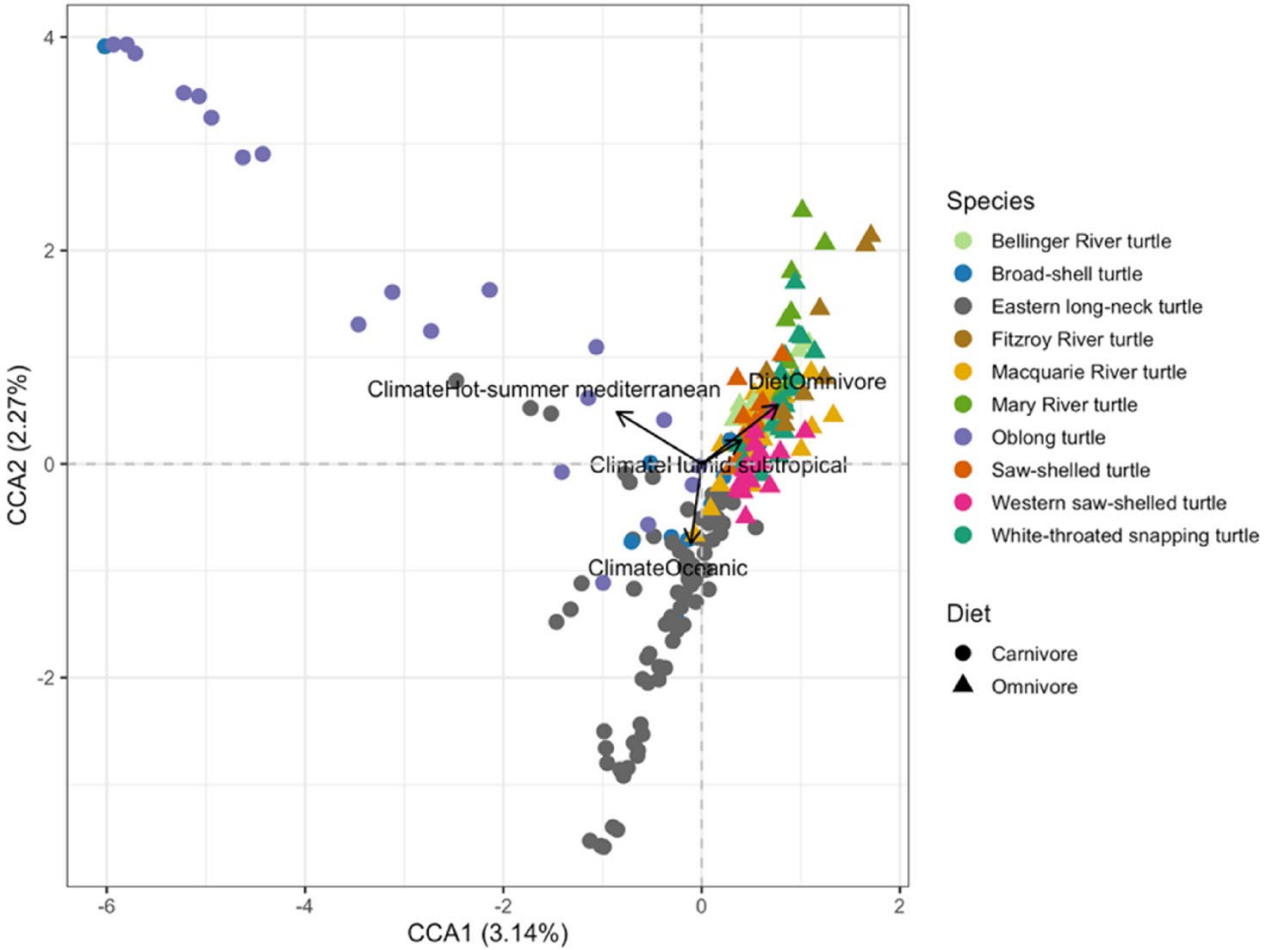
Canonical correspondence analysis (CCA) showing relationships between bacterial community composition at genus level, host diet and climatic variables. Each point represents an individual sample, coloured by species and shaped based on dietary category.

Canonical Correspondence Analysis revealed that diet and climate significantly influenced bacterial community composition (df = 4, *χ* ^2^ = 0.87, f.model = 5.15, *p* < 0.01) (Figure 4). A marginal permutation test confirmed that both diet (df = 1, *χ* ^2^ = 0.19, f.model = 4.45, *p* < 0.01) and climate (df = 3, *χ* ^2^ = 0.57, f.model = 4.47, *p* < 0.01) were highly significant drivers of microbial community composition. These variables collectively explained approximately 7.6% of the total variation in the data, indicating that while they are important factors, a large proportion of community variability remains unexplained by the variables included in this model.

**FIGURE 5:**
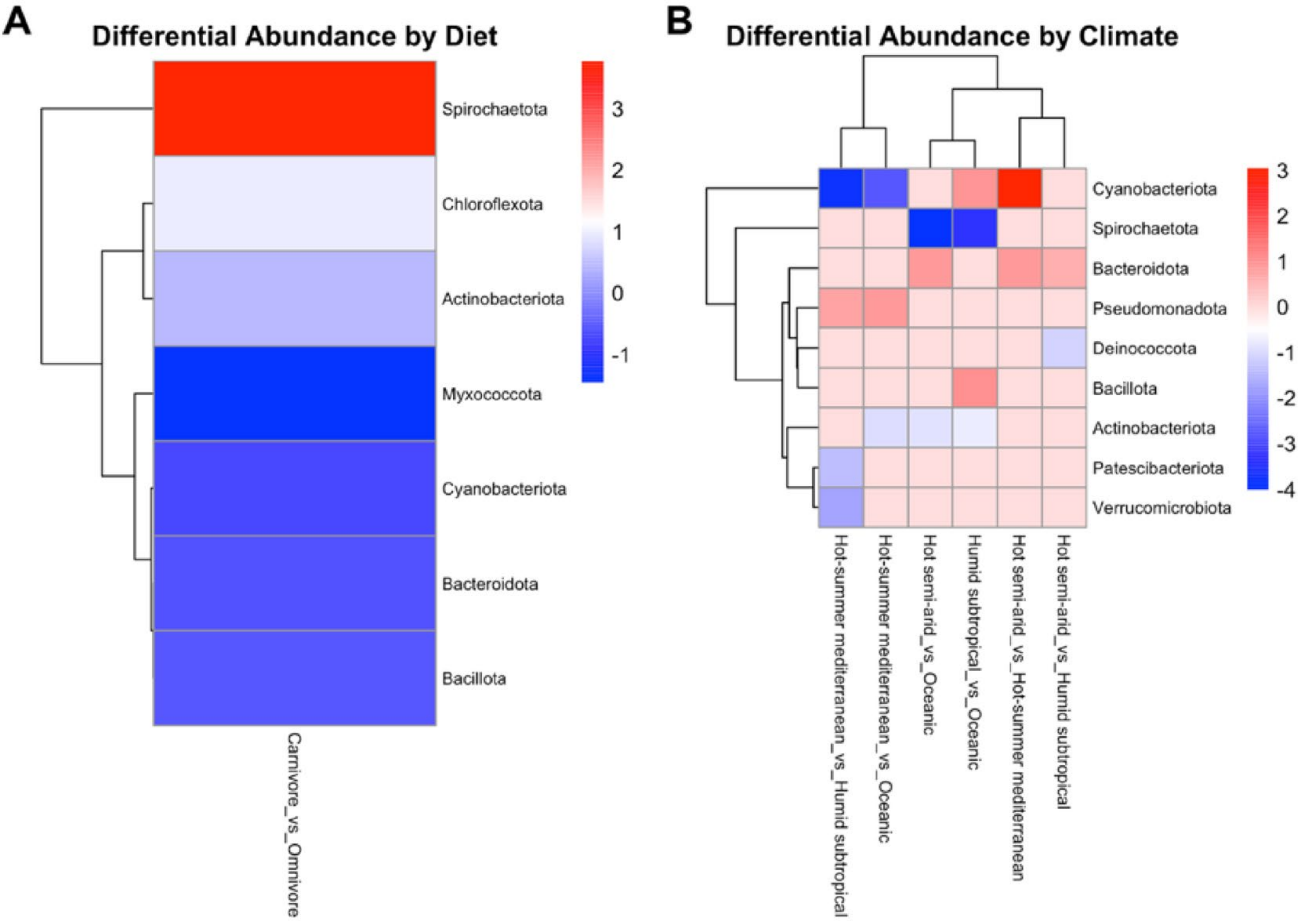
Differential abundance of bacterial phyla associated with climate and diet. Heatmaps display the log2 fold change of significantly differentially abundant bacterial phyla, as determined by pairwise comparisons using DESeq2 for climate (A) and diet (B). In both heatmaps, rows represent bacterial phyla that were significantly different in at least one comparison. The colour intensity and scale indicate the magnitude of the log2 fold change. Red hues denote enrichment in the first group of a comparison pair, while blue hues denote enrichment in the second. Phyla in each panel are clustered hierarchically based on their log2 fold change patterns.

Differential abundance testing revealed that the phyla Bacteroidota (log2 Fold Change = +0.58, Adjusted *p* < 0.01), Myxococcota (+1.46, Adjusted *p* < 0.01), Cyanobacteriota (+0.70, Adjusted *p* < 0.01) and Bacillota (+0.57, Adjusted *p* < 0.01) were significantly more abundant in the microbiota of omnivorous turtles (Table S9). A differential abundance analysis using DESeq2 revealed several significant differences in phylum‐level composition among the four climate zones: hot semi‐arid, hot‐summer mediterranean, humid subtropical and oceanic. Our pairwise comparisons identified distinct phyla enriched in each climate. For instance, turtles from hot semi‐arid regions showed enrichment of Cyanobacteriota and Bacteroidota compared to those from hot‐summer mediterranean zones but were also characterised by a higher abundance of Bacteroidota relative to humid subtropical zones. Turtles in oceanic climates exhibited higher levels of Spirochaetota and Actinobacteriota than those from hot semi‐arid regions, while experiencing lower abundance of Cyanobacteriota, Pseudomonadota and Actinobacteriota compared to hot‐summer mediterranean zones. Conversely, humid subtropical climates were associated with lower levels of Spirochaetota and Actinobacteriota relative to oceanic climates, but higher levels of Cyanobacteriota. These findings indicate that climatic variation is a strong determinant of the turtle gut microbiota, driving distinct compositional shifts at the phylum level. A complete summary of all pairwise comparisons and the associated log2 fold changes and adjusted *p*‐values is detailed in Table S10 and the relative abundance of significantly different phyla across climate groups is visually depicted in Figure 5.

When we combined host phylogeny into our dataset, analysis revealed a significant relationship between evolutionary history and microbiota composition in freshwater turtles, providing statistical evidence for phylosymbiosis within this group. The Mantel test demonstrated a weak but highly significant correlation between host phylogenetic distance and microbiota community dissimilarity (Mantel *r* = 0.12, *p* < 0.01), indicating that more closely related turtle species tend to harbour more similar microbiotas. Consistent with this finding, PERMANOVA confirmed that host species is a significant factor shaping microbiota structure (df = 9, SS_T_ = 6.37, *R* ^2^ = 0.05, f.model = 1.46, *p* < 0.01). Importantly, the overall dbRDA model was statistically significant (df = 2, SS_T_ = 1.04, f.model = 1.3, *p* = 0.01), indicating that host evolutionary history contributes significantly to variation in gut microbial communities of turtle species. However, effect sizes were small, with host species explaining only 5.06% of the total variation. Diet and climate together explained 7.6% of variation, suggesting that while these factors are statistically significant, each accounts for a relatively modest proportion of overall community variation. Collectively, these results indicate that turtle gut microbiotas are structured by multiple weak but significant influences rather than a single dominant driver.

**FIGURE 6:**
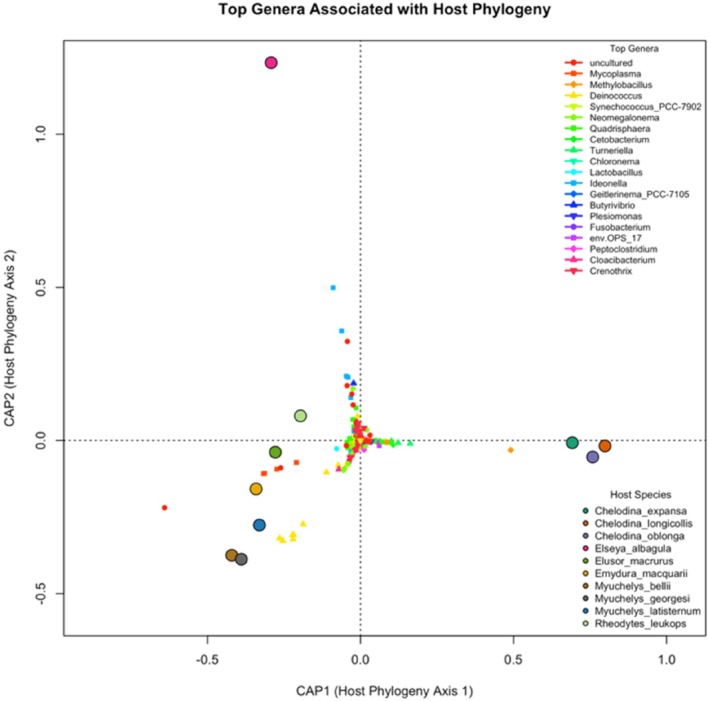
Distance‐based redundancy analysis (dbRDA) ordination visualising the relationship between host phylogeny and microbiota community composition. The axes represent constrained (CAP1) and unconstrained (CAP2) variation. The plot highlights the top 20 genera contributing most significantly to the host‐associated variation using coloured points mapped via a synchronised legend.

**FIGURE 7:**
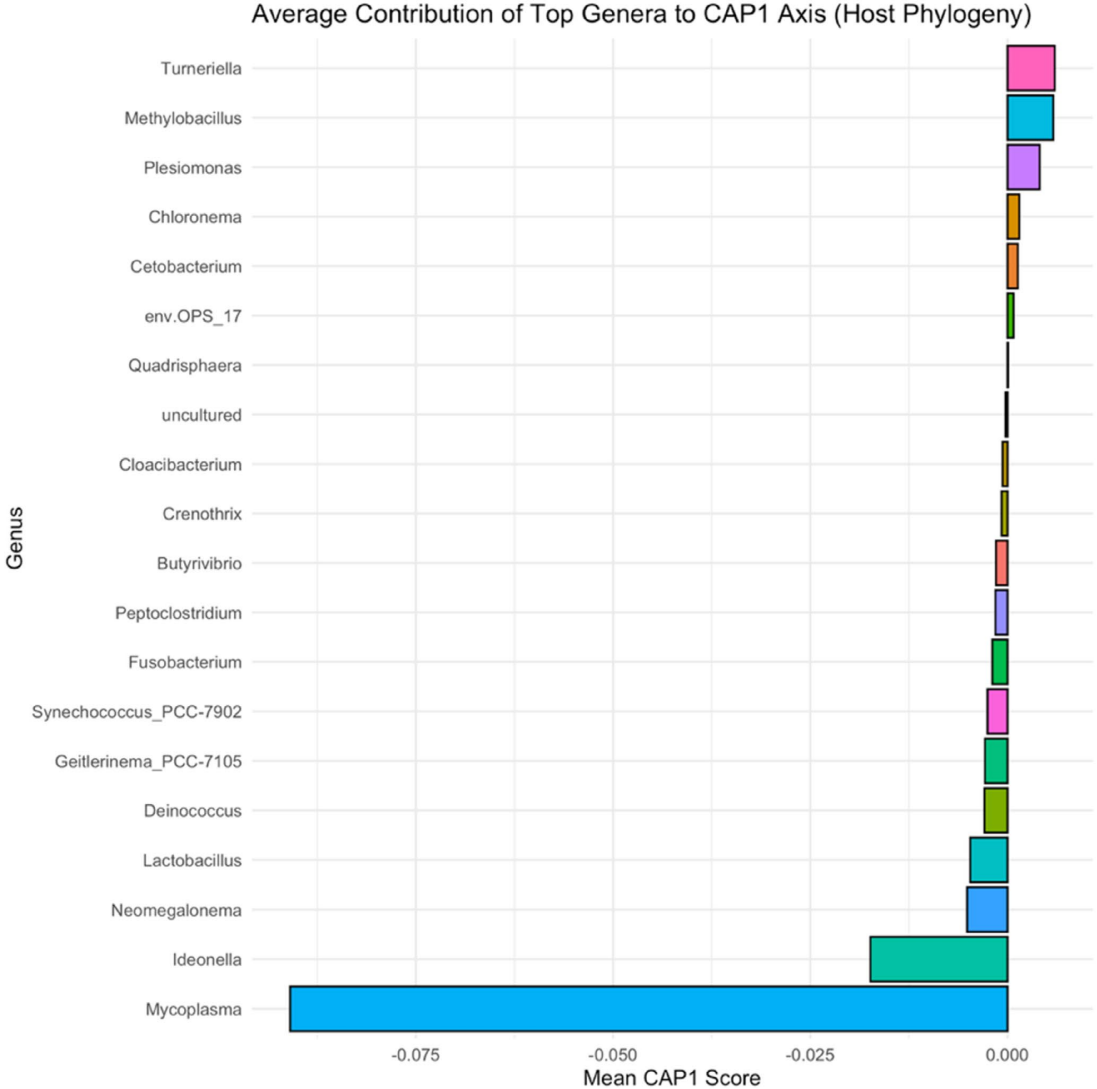
Average contribution of the top 20 most abundant genera to the dbRDA CAP1 axis. The bar plot displays the mean CAP1 score for each genus. Positive Mean CAP1 score (bars extending to the right) represents genera that correlate positively with the host phylogeny axis (CAP1), indicating a strong association with specific host lineages captured by that axis. Negative Mean CAP1 Score (bars extending left) indicates these genera are associated with the host lineages that plot on the negative side of the CAP1 axis.

The two primary constrained axes of the model (CAP1 and CAP2) are presented in the ordination plot (Figure 6). This visualisation highlights the separation of microbial communities along the primary host phylogeny axis and identifies the top 20 genera contributing to this pattern with all scores available in Additional File 2. Furthermore, a bar graph was generated (Figure 7) to display the average CAP1 scores for the top 20 genera. Genera with positive CAP1 scores are positively associated with one end of the host phylogenetic gradient, while those with negative scores are associated with the opposite end. These results demonstrate specific microbial taxa that covary significantly with the host phylogeny.

## 4 Discussion

This study provides the most extensive analysis to date of the cloacal microbiota of Australian freshwater turtles, representing 10 species sampled across diverse climatic zones and ecological contexts. By integrating host phylogeny, diet and environmental variables, we identified key factors shaping microbial diversity and structure within this ancient reptilian lineage. Our results reveal that host evolutionary history exerts a measurable influence on microbial composition and additionally ecological factors, particularly diet and climate, also play a substantial role in determining microbiota assembly. Together, these findings advance understanding of reptilian microbiotas and illuminate the complex interplay between evolutionary heritage and environmental adaptation in shaping host‐associated microbial communities. Across all individuals, the cloacal microbiota of Australian freshwater turtles was taxonomically rich, encompassing 38 bacterial phyla and nearly 3000 unique ASVs. The dominance of Pseudomonadota, Actinobacteriota, Bacteroidota, Deinococcota and Bacillota mirrors patterns seen in other chelonians (Scheelings et al. 2020; Jenkins et al. 2024; Zakaria et al. 2025; Blair et al. 2025; Wu et al. 2021), suggesting the existence of a broadly conserved microbial consortium associated with this taxon. The enrichment of Bacteroidota in omnivorous turtles may be particularly important given the central role of many members of this phylum, including *Bacteroides* spp., in the degradation of complex carbohydrates and the production of short‐chain fatty acids that contribute to host nutrition (Shin et al. 2024). The widespread occurrence of Bacteroidota as dominant gut symbionts across vertebrate taxa suggests that these bacteria may represent evolutionarily important host‐associated microbes. Although our data do not allow assessment of co‐evolutionary relationships across a broad spectrum of chelonian species, the consistent association of these taxa with turtle gastrointestinal tracts highlights them as promising candidates for future studies investigating host–microbe coadaptation and holobiont dynamics.

Microbial alpha diversity varied significantly among turtle species, diets and climatic zones. Differences among sites and species likely reflect the combined influence of habitat type, water quality and available microbial reservoirs. Australian freshwater turtles in this study were sampled from a range of natural freshwater habitats that can vary in hydrology, turbidity and organic matter load (von Schiller et al. 2017). Although these environmental variables were not explicitly quantified in this study, in future investigations these parameters should be measured and incorporated in data analysis to contextualise their impact on shaping chelonian microbiota diversity and composition.

Dietary variation emerged as a key determinant of microbiota diversity in Australian freshwater turtles. Carnivorous species exhibited higher observed ASV richness and Shannon diversity than omnivorous counterparts, a pattern contrasting with trends in mammals and birds, where omnivores typically host more diverse microbial consortia capable of metabolising varied substrates (Scheelings et al. 2020; von Schiller et al. 2017). However, similar patterns reported in ectothermic vertebrates, particularly fish, where diet is a strong driver of gut microbial diversity, with carnivory often associated with distinct and sometimes more diverse microbial assemblages (Sullam et al. 2012). In turtles, this increased microbial richness may reflect transient colonisation by prey‐associated microbes and specialisation for proteolytic and fermentative metabolism, suggesting that microbial diversity in carnivores is shaped more by ecological exposure and substrate complexity than by generalist foraging strategies. Taxonomic analyses further revealed that omnivorous turtles were enriched in Bacteroidota, Myxococcota, Cyanobacteriota and Bacillota, which have been shown to play central roles in carbohydrate breakdown and short‐chain fatty acid production, facilitating nutrient extraction from detrital and algal material (Pardesi et al. 2022). Enrichment of fermentative and degradative taxa in omnivores likely enhances digestive flexibility, whereas carnivorous turtles may rely on microbial guilds specialised for protein fermentation and amino acid metabolism, paralleling dietary–microbiota relationships observed in other reptiles such as lizards (Hong et al. 2011). However, these interpretations should be considered cautiously because diet and phylogeny were strongly confounded in our dataset. All carnivorous species belonged to the genus *Chelodina*, whereas the omnivorous species represented more distantly related lineages that diverged approximately 70 million years ago (Figure 1). Consequently, the observed differences in microbial diversity and composition may reflect dietary effects, phylogenetic relatedness or an interaction between the two. Future studies incorporating a broader range of diets are not possible with Australian species alone, as there are no herbivorous pleurodiran chelonians in Australia and would need to incorporate a broader taxonomic breadth to disentangle these influences.

Climatic gradients exerted a strong influence on microbial diversity and composition across Australian freshwater turtles. Turtles inhabiting oceanic and humid subtropical zones exhibited higher microbial richness and evenness than those from semi‐arid and Mediterranean climates, reflecting broader environmental microbial pools and more stable hydrological conditions. Warmer, wetter regions support greater environmental microbial diversity, providing a richer source for colonisation (Barnard et al. 2015), whereas arid and thermally variable habitats impose physiological and ecological constraints that limit microbial persistence (Maestre et al. 2015). These results align with patterns reported in other vertebrates, where external conditions directly shape microbial exposure and colonisation dynamics (Sepulveda and Moeller 2020; Moeller et al. 2020).

Community‐level analyses revealed clear differences in microbiota composition among turtle species, dietary groups and climatic zones, although the strength of these effects varied among factors. The strong signal observed for weighted and unweighted UniFrac metrics suggests that both rare and abundant taxa contribute substantially to between‐host variation. This pattern indicates that subtle ecological or physiological differences among species may influence the recruitment and persistence of microbial lineages, even within a broadly conserved core microbiota among related hosts. Similar trends have been reported in amphibians, where low‐abundant taxa often reflect environmental exposure and host‐specific filtering processes (Bletz et al. 2016). The growing recognition that rare taxa can shape functional potential and ecosystem resilience supports the idea that they play disproportionate roles in maintaining microbial stability under environmental stress (Chakraborty et al. 2025).

Canonical Correspondence Analysis confirmed that both diet and climate significantly predicted bacterial community structure, jointly explaining 2.76% of total variation. Although modest, this level of explained variance is consistent with other host–microbiota studies, where stochastic colonisation, horizontal transfer and unmeasured host traits contribute substantial unexplained variability (Parfrey et al. 2018). Importantly, the ordination revealed ecological segregation of microbial assemblages along dietary and climatic gradients, supporting the hypothesis that multiple selective forces interact to shape microbial community assembly in freshwater turtles.

Beyond ecology, host evolutionary history also contributed to microbiota variation, albeit modestly. The Mantel test identified a weak but significant correlation (*r* = 0.12) between host phylogenetic distance and microbiota dissimilarity, consistent with the concept of phylosymbiosis, where host evolutionary relationships are mirrored in microbial community composition (Scheelings et al. 2020). The weak magnitude of phylosymbiosis observed here likely reflects overlapping geographic distributions, shared aquatic environments and conserved digestive anatomies among Australian freshwater turtles. Their semi‐aquatic lifestyle promotes continual microbial exchange with the environment, diluting species‐specific microbial signatures. Additionally, many chelid turtles exhibit generalist feeding habits, reducing dietary niche differentiation that could otherwise drive microbial divergence. Together, these findings suggest that while host evolutionary history exerts a detectable influence on microbiota assembly, ecological plasticity and environmental connectivity are stronger determinants in this system.

The significant association found between host phylogeny and microbiome community structure suggests a pattern of phylosymbiosis within Australian freshwater turtles, where evolutionary relatedness among hosts predicts similarities in their gut microbial composition. The dbRDA identified key microbial drivers of this association, primarily dominated by members of the Pseudomonadota, including *Methylobacillus* and the Actinobacteriota (uncultured genera). The strong positive correlation of these taxa with the CAP1 axis indicates they are crucial differentiating features among host lineages. Genera such as *Turneriella* (Spirochaetota), *Deinococcus* (Deinococcota) and *Cetobacterium* (Fusobacteriota) were also among the top contributors, suggesting their presence or absence is structured by host evolutionary history. These patterns indicate that host‐associated factors, whether diet, genetics, morphology or gut physiology, selectively favour these specific microbial communities, likely facilitating long‐term co‐diversification or host specialisation. One potential mechanism underlying the phylosymbiotic patterns observed here is the vertical transmission of host‐associated microbes across generations. If certain bacterial taxa are consistently transferred from females to offspring, they may become associated with host evolutionary lineages and contribute to concordance between host phylogeny and microbiome composition. However, because microbial transmission was not assessed in this study, alternative mechanisms such as environmental acquisition, host physiological filtering and shared ecological traits cannot be excluded. Future studies examining microbial communities in eggs, hatchlings and adults will be important for determining the role of vertical transmission in shaping turtle gut microbiomes.

As climate change intensifies across Australia, freshwater habitats are undergoing rapid transformation resulting in declines in water quantity and quality (Pittock and Finlayson 2011). These environmental changes are likely to reshape microbial assemblages both within and around turtle populations. Given that microbiotas can respond to environmental perturbations more rapidly than host genomes, they may act as early indicators of ecosystem stress and potential determinants of host resilience. Integrating microbiota monitoring into conservation frameworks could thus improve our capacity to predict population responses to environmental change (Trevelline et al. 2019).

Nearly half of Australia's freshwater turtle species are currently threatened due to habitat degradation, invasive predators and emerging diseases (Van Dyke et al. 2018). Incorporating microbiota perspectives into conservation and rehabilitation practices may offer novel management opportunities. Likewise, baseline microbial profiles could serve as biomarkers of environmental quality or physiological stress. Recognising the microbiota as a component of host biology is thus essential for holistic, evidence‐based conservation of freshwater turtles in an era of rapid global change.

## 5 Conclusions

In summary, our study demonstrates that the microbiotas of Australian freshwater turtles are structured by a combination of diet, climate and evolutionary history. While host phylogeny exerts a detectable influence, ecological factors appear to play a dominant role in shaping microbial communities. These findings highlight the value of using a holistic, multi‐factor approach to understanding host–microbiota evolution in ectothermic vertebrates and provide an important foundation for future studies exploring the functional and conservation relevance of microbial symbioses in threatened freshwater turtles.

## Author Contributions

**Thi Thu Hao Van:** writing – original draft, methodology, validation, visualization, writing – review and editing, software, data curation. **Lee F. Skerratt:** investigation, methodology, funding acquisition, writing – original draft, writing – review and editing, supervision, resources, project administration. **Anthony Santoro:** investigation, methodology, writing – review and editing, formal analysis, resources, validation. **Robert J. Moore:** investigation, funding acquisition, writing – original draft, methodology, writing – review and editing, supervision, resources, data curation. **T. Franciscus Scheelings:** conceptualization, investigation, writing – original draft, methodology, validation, writing – review and editing, formal analysis, project administration. **James U. Van Dyke:** investigation, methodology, validation, writing – review and editing, formal analysis, resources. **Christopher T. Ormond:** investigation, methodology, validation, writing – review and editing, formal analysis, resources. **Louise M. Streeting:** investigation, methodology, writing – review and editing, formal analysis, resources, validation.

## Ethics Statement

This study was approved by The University of Melbourne Office of Research Ethics and Integrity (Ethics ID: 2022‐24808‐32226‐4) and all experiments were performed in accordance with relevant guidelines and regulations.

## Conflicts of Interest

The authors declare no conflicts of interest.

## Supporting information


**Table S1:** Pairwise comparisons for Observed ASVs for Australian freshwater turtles when analysed for species (bold numbers indicate significant results, with all *p*‐values having been adjusted with Bonferroni correction).
**Table S2:** Pairwise comparisons for Inverse Simpson for Australian freshwater turtles when analysed for species (bold numbers indicate significant results, with all *p*‐values having been adjusted with Bonferroni correction).
**Table S3:** Pairwise comparisons for Shannon for Australian freshwater turtles when analysed for species (bold numbers indicate significant results, with all *p*‐values having been adjusted with Bonferroni correction).
**Table S4:** Pairwise comparisons for Observed ASVs for Australian freshwater turtles when analysed for climate (bold numbers indicate significant results, with all *p*‐values having been adjusted with Bonferroni correction).
**Table S5:** Pairwise comparisons for Inverse Simpson for Australian freshwater turtles when analysed for climate (bold numbers indicate significant results, with all *p*‐values having been adjusted with Bonferroni correction).
**Table S6:** Pairwise comparisons for Shannon for Australian freshwater turtles when analysed for climate (bold numbers indicate significant results, with all *p*‐values having been adjusted with Bonferroni correction).
**Table S7:** Pairwise comparisons for weighted UniFrac for Australian freshwater turtles (bold values indicate significant results).
**Table S8:** Pairwise comparisons for unweighted UniFrac for Australian freshwater turtles (bold values indicate significant results).
**Table S9:** Results of differential abundance testing for bacterial phyla and diet in Australian freshwater turtles (bold values indicate significant results).
**Table S10:** Results of differential abundance testing for bacterial phyla and climate in Australian freshwater turtles (bold values indicate significant results).

**Data S1:** emi70396‐sup‐0002‐Supinfo2.csv.

## Data Availability

The datasets generated and/or analysed during the current study are available in *The National Center for Biotechnology Information* www.ncbi.nlm.nih.gov (PRJNA1478026) and may be made available on reasonable request from the authors.
