# Supplementary material for: From Genes to Greens: How Diet, Climate and Phylogeny Shape Freshwater Turtle Microbiotas
Source: Environ Microbiol. 2026 Aug 6;28(8):e70396. doi: 10.1111/1462-2920.70396 (PMC13446997; doi:10.1111/1462-2920.70396)
Supplement: Supplementary file 1 [file EMI-28-e70396-s001.docx]

**Additional File**

**Abbreviations:**

Bellinger River turtle = BRT

Eastern long-neck turtle = ELNT

Fitzroy River Turtle = FRT

Macquarie River turtle = MacRT

Mary River turtle = MRT

Western saw-shelled turtle = WSST

Oblong turtle = OT

Saw-shelled turtle = SST

White-throated snapping turtle = WTST

**Table S1.** Pairwise comparisons for Observed ASVs for Australian freshwater turtles when analysed for species (bold numbers indicate significant results, with all p-values having been adjusted with Bonferroni correction).

| Species | BRT | BST | ELNT | FRT | MacRT | MRT | OT | SST | WSST |
| --- | --- | --- | --- | --- | --- | --- | --- | --- | --- |
| BST | **0.03** | - | - | - | - | - | - | - | - |
| ELNT | **<0.01** | 1.00 | - | - | - | - | - | - | - |
| FRT | 1.00 | 1.00 | 1.00 | - | - | - | - | - | - |
| MacRT | **<0.01** | 1.00 | **<0.01** | 1.00 | - | - | - | - | - |
| MRT | 1.00 | 1.00 | 0.26 | 1.00 | 1.00 | - | - | - | - |
| OT | **0.02** | 1.00 | 0.78 | 1.00 | 1.00 | 1.00 | - | - | - |
| SST | **<0.01** | 1.00 | 1.00 | 1.00 | 0.56 | 0.72 | 1.00 | - | - |
| WSST | **<0.01** | 0.66 | 0.30 | 1.00 | **<0.01** | **0.02** | **0.02** | 0.56 | - |
| WTST | **<0.01** | 1.00 | 1.00 | 1.00 | 1.00 | 1.00 | 1.00 | 1.00 | 0.22 |

**Table S2.** Pairwise comparisons for Inverse Simpson for Australian freshwater turtles when analysed for species (bold numbers indicate significant results, with all p-values having been adjusted with Bonferroni correction).

| Species | BRT | BST | ELNT | FRT | MacRT | MRT | OT | SST | WSST |
| --- | --- | --- | --- | --- | --- | --- | --- | --- | --- |
| BST | **0.03** | - | - | - | - | - | - | - | - |
| ELNT | **<0.01** | 1.00 | - | - | - | - | - | - | - |
| FRT | 1.00 | 1.00 | 1.00 | - | - | - | - | - | - |
| MacRT | **<0.01** | 1.00 | **<0.01** | 1.00 | - | - | - | - | - |
| MRT | 1.00 | 1.00 | 0.26 | 1.00 | 1.00 | - | - | - | - |
| OT | **0.02** | 1.00 | 0.78 | 1.00 | 1.00 | 1.00 | - | - | - |
| SST | **<0.01** | 1.00 | 1.00 | 1.00 | 0.56 | 0.72 | 1.00 | - | - |
| WSST | **<0.01** | 0.66 | 0.30 | 1.00 | **<0.01** | **0.02** | **0.02** | 0.56 | - |
| WTST | **<0.01** | 1.00 | 1.00 | 1.00 | 1.00 | 1.00 | 1.00 | 1.00 | 0.22 |

**Table S3.** Pairwise comparisons for Shannon for Australian freshwater turtles when analysed for species (bold numbers indicate significant results, with all p-values having been adjusted with Bonferroni correction).

| Species | BRT | BST | ELNT | FRT | MacRT | MRT | OT | SST | WSST |
| --- | --- | --- | --- | --- | --- | --- | --- | --- | --- |
| BST | **0.03** | - | - | - | - | - | - | - | - |
| ELNT | **<0.01** | 1.00 | - | - | - | - | - | - | - |
| FRT | 1.00 | 1.00 | 1.00 | - | - | - | - | - | - |
| MacRT | **<0.01** | 1.00 | **<0.01** | 1.00 | - | - | - | - | - |
| MRT | 1.00 | 1.00 | 0.26 | 1.00 | 1.00 | - | - | - | - |
| OT | **0.02** | 1.00 | 0.78 | 1.00 | 1.00 | 1.00 | - | - | - |
| SST | **<0.01** | 1.00 | 1.00 | 1.00 | 0.56 | 0.72 | 1.00 | - | - |
| WSST | **<0.01** | 0.66 | 0.30 | 1.00 | **<0.01** | **0.02** | **0.02** | 0.56 | - |
| WTST | **<0.01** | 1.00 | 1.00 | 1.00 | 1.00 | 1.00 | 1.00 | 1.00 | 0.22 |

**Table S4.** Pairwise comparisons for Observed ASVs for Australian freshwater turtles when analysed for climate (bold numbers indicate significant results, with all p-values having been adjusted with Bonferroni correction).

| Climate | Hot semi-arid | Hot-summer mediterranean | Humid subtropical |
| --- | --- | --- | --- |
| Hot-summer mediterranean | 1.00 | - | - |
| Humid subtropical | 0.25 | 1.00 | - |
| Oceanic | **<0.01** | 0.11 | **0.01** |

**Table S5.** Pairwise comparisons for Inverse Simpson for Australian freshwater turtles when analysed for climate (bold numbers indicate significant results, with all p-values having been adjusted with Bonferroni correction).

| Climate | Hot semi-arid | Hot-summer mediterranean | Humid subtropical |
| --- | --- | --- | --- |
| Hot-summer mediterranean | **<0.01** | - | - |
| Humid subtropical | 1.00 | **<0.01** | - |
| Oceanic | 1.00 | **<0.01** | 1.00 |

**Table S6.** Pairwise comparisons for Shannon for Australian freshwater turtles when analysed for climate (bold numbers indicate significant results, with all p-values having been adjusted with Bonferroni correction).

| Climate | Hot semi-arid | Hot-summer mediterranean | Humid subtropical |
| --- | --- | --- | --- |
| Hot-summer mediterranean | **0.05** | - | - |
| Humid subtropical | 1.00 | **<0.01** | - |
| Oceanic | 0.82 | **<0.01** | 1.00 |

**Table S7.** Pairwise comparisons for weighted UniFrac for Australian freshwater turtles (bold values indicate significant results)

| Pairs | df | SS_T_ | f.model | R^2^ | p | p.adjust |
| --- | --- | --- | --- | --- | --- | --- |
| BST vs ELNT | 1.00 | 0.26 | 2.82 | 0.03 | <0.01 | 0.14 |
| BST vs MacRT | 1.00 | 0.39 | 4.89 | 0.08 | <0.01 | **0.05** |
| BST vs OT | 1.00 | 0.45 | 4.94 | 0.15 | <0.01 | 0.09 |
| BST vs BRT | 1.00 | 0.42 | 6.25 | 0.13 | <0.01 | **0.05** |
| BST vs WTST | 1.00 | 0.28 | 3.37 | 0.10 | <0.01 | **0.05** |
| BST vs MRT | 1.00 | 0.63 | 6.82 | 0.29 | <0.01 | **0.05** |
| BST vs SST | 1.00 | 0.24 | 3.91 | 0.15 | <0.01 | **0.05** |
| BST vs WSST | 1.00 | 0.21 | 2.95 | 0.10 | <0.01 | 0.09 |
| BST vs FRT | 1.00 | 0.24 | 2.82 | 0.14 | 0.03 | 1.00 |
| ELNT vs MacRT | 1.00 | 1.22 | 13.88 | 0.10 | <0.01 | **0.05** |
| ELNT vs OT | 1.00 | 1.03 | 11.05 | 0.10 | <0.01 | **0.05** |
| ELNT vs BRT | 1.00 | 0.83 | 9.87 | 0.08 | <0.01 | **0.05** |
| ELNT vs WTST | 1.00 | 0.68 | 7.51 | 0.07 | <0.01 | **0.05** |
| ELNT vs MRT | 1.00 | 1.01 | 10.82 | 0.11 | <0.01 | **0.05** |
| ELNT vs SST | 1.00 | 0.49 | 5.76 | 0.06 | <0.01 | **0.05** |
| ELNT vs WSST | 1.00 | 0.45 | 5.18 | 0.05 | <0.01 | **0.05** |
| ELNT vs FRT | 1.00 | 0.52 | 5.67 | 0.06 | <0.01 | **0.05** |
| MacRT vs OT | 1.00 | 1.20 | 14.17 | 0.18 | <0.01 | **0.05** |
| MacRT vs BRT | 1.00 | 0.51 | 7.07 | 0.08 | <0.01 | **0.05** |
| MacRT vs WTST | 1.00 | 0.90 | 11.23 | 0.14 | <0.01 | **0.05** |
| MacRT vs MRT | 1.00 | 1.00 | 12.10 | 0.18 | <0.01 | **0.05** |
| MacRT vs SST | 1.00 | 0.61 | 8.42 | 0.12 | <0.01 | **0.05** |
| MacRT vs WSST | 1.00 | 0.32 | 4.22 | 0.06 | <0.01 | **0.05** |
| MacRT vs FRT | 1.00 | 0.64 | 7.86 | 0.13 | <0.01 | **0.05** |
| OT vs BRT | 1.00 | 1.06 | 14.10 | 0.22 | <0.01 | **0.05** |
| OT vs WTST | 1.00 | 0.79 | 8.93 | 0.19 | <0.01 | **0.05** |
| OT vs MRT | 1.00 | 1.00 | 10.16 | 0.28 | <0.01 | **0.05** |
| OT vs SST | 1.00 | 0.73 | 9.68 | 0.23 | <0.01 | **0.05** |
| OT vs WSST | 1.00 | 0.73 | 8.92 | 0.20 | <0.01 | **0.05** |
| OT vs FRT | 1.00 | 0.32 | 3.39 | 0.12 | 0.01 | 0.45 |
| BRT vs WTST | 1.00 | 0.62 | 8.86 | 0.15 | <0.01 | **0.05** |
| BRT vs MRT | 1.00 | 0.90 | 12.92 | 0.25 | <0.01 | **0.05** |
| BRT vs SST | 1.00 | 0.40 | 6.92 | 0.14 | <0.01 | **0.05** |
| BRT vs WSST | 1.00 | 0.40 | 6.39 | 0.12 | <0.01 | **0.05** |
| BRT vs FRT | 1.00 | 0.59 | 8.74 | 0.19 | <0.01 | **0.05** |
| WTST vs MRT | 1.00 | 0.68 | 7.76 | 0.22 | <0.01 | **0.05** |
| WTST vs SST | 1.00 | 0.21 | 3.12 | 0.09 | <0.01 | 0.18 |
| WTST vs WSST | 1.00 | 0.39 | 5.26 | 0.13 | <0.01 | **0.05** |
| WTST vs FRT | 1.00 | 0.22 | 2.65 | 0.09 | 0.02 | 0.99 |
| MRT vs SST | 1.00 | 0.65 | 9.83 | 0.33 | <0.01 | **0.05** |
| MRT vs WSST | 1.00 | 0.86 | 11.26 | 0.33 | <0.01 | **0.05** |
| MRT vs FRT | 1.00 | 0.66 | 6.78 | 0.33 | <0.01 | **0.05** |
| SST vs WSST | 1.00 | 0.26 | 4.71 | 0.14 | <0.01 | **0.05** |
| SST vs FRT | 1.00 | 0.35 | 5.75 | 0.22 | <0.01 | **0.05** |
| WSST vs FRT | 1.00 | 0.36 | 4.98 | 0.18 | <0.01 | **0.05** |

**Table S8.** Pairwise comparisons for unweighted UniFrac for Australian freshwater turtles (bold values indicate significant results)

| Pairs | df | SS_T_ | f.model | R^2^ | p | p.adjust |
| --- | --- | --- | --- | --- | --- | --- |
| BST vs ELNT | 1.00 | 0.44 | 2.87 | 0.03 | <0.01 | 0.09 |
| BST vs MacRT | 1.00 | 0.57 | 3.74 | 0.06 | <0.01 | **0.05** |
| BST vs OT | 1.00 | 1.37 | 9.42 | 0.25 | <0.01 | **0.05** |
| BST vs BRT | 1.00 | 1.19 | 8.47 | 0.17 | <0.01 | **0.05** |
| BST vs WTST | 1.00 | 0.58 | 3.48 | 0.10 | <0.01 | **0.05** |
| BST vs MRT | 1.00 | 0.60 | 3.44 | 0.17 | <0.01 | **0.05** |
| BST vs SST | 1.00 | 0.74 | 6.01 | 0.21 | <0.01 | **0.05** |
| BST vs WSST | 1.00 | 0.67 | 5.67 | 0.18 | <0.01 | **0.05** |
| BST vs FRT | 1.00 | 0.47 | 2.60 | 0.13 | <0.01 | 0.09 |
| ELNT vs MacRT | 1.00 | 2.29 | 15.43 | 0.11 | <0.01 | **0.05** |
| ELNT vs OT | 1.00 | 2.86 | 19.66 | 0.17 | <0.01 | **0.05** |
| ELNT vs BRT | 1.00 | 3.95 | 27.43 | 0.20 | <0.01 | **0.05** |
| ELNT vs WTST | 1.00 | 1.90 | 12.47 | 0.11 | <0.01 | **0.05** |
| ELNT vs MRT | 1.00 | 1.25 | 8.21 | 0.09 | <0.01 | **0.05** |
| ELNT vs SST | 1.00 | 1.72 | 12.23 | 0.12 | <0.01 | **0.05** |
| ELNT vs WSST | 1.00 | 1.61 | 11.66 | 0.11 | <0.01 | **0.05** |
| ELNT vs FRT | 1.00 | 1.05 | 6.86 | 0.08 | <0.01 | **0.05** |
| MacRT vs OT | 1.00 | 2.12 | 15.05 | 0.19 | <0.01 | **0.05** |
| MacRT vs BRT | 1.00 | 1.25 | 8.98 | 0.10 | <0.01 | **0.05** |
| MacRT vs WTST | 1.00 | 0.79 | 5.25 | 0.07 | <0.01 | **0.05** |
| MacRT vs MRT | 1.00 | 0.56 | 3.73 | 0.06 | <0.01 | **0.05** |
| MacRT vs SST | 1.00 | 0.86 | 6.50 | 0.10 | <0.01 | **0.05** |
| MacRT vs WSST | 1.00 | 1.02 | 7.90 | 0.11 | <0.01 | **0.05** |
| MacRT vs FRT | 1.00 | 0.71 | 4.67 | 0.08 | <0.01 | **0.05** |
| OT vs BRT | 1.00 | 1.98 | 15.45 | 0.24 | <0.01 | **0.05** |
| OT vs WTST | 1.00 | 1.90 | 13.12 | 0.25 | <0.01 | **0.05** |
| OT vs MRT | 1.00 | 1.02 | 7.24 | 0.22 | <0.01 | **0.05** |
| OT vs SST | 1.00 | 1.48 | 13.54 | 0.30 | <0.01 | **0.05** |
| OT vs WSST | 1.00 | 2.49 | 23.42 | 0.40 | <0.01 | **0.05** |
| OT vs FRT | 1.00 | 1.32 | 9.17 | 0.26 | <0.01 | **0.05** |
| BRT vs WTST | 1.00 | 1.23 | 8.69 | 0.15 | <0.01 | **0.05** |
| BRT vs MRT | 1.00 | 0.80 | 5.84 | 0.13 | <0.01 | **0.05** |
| BRT vs SST | 1.00 | 1.41 | 12.29 | 0.22 | <0.01 | **0.05** |
| BRT vs WSST | 1.00 | 1.94 | 17.28 | 0.27 | <0.01 | **0.05** |
| BRT vs FRT | 1.00 | 0.94 | 6.70 | 0.15 | <0.01 | **0.05** |
| WTST vs MRT | 1.00 | 0.45 | 2.71 | 0.09 | <0.01 | 0.14 |
| WTST vs SST | 1.00 | 0.46 | 3.57 | 0.10 | <0.01 | **0.05** |
| WTST vs WSST | 1.00 | 0.78 | 6.21 | 0.15 | <0.01 | **0.05** |
| WTST vs FRT | 1.00 | 0.33 | 1.98 | 0.07 | 0.01 | 0.54 |
| MRT vs SST | 1.00 | 0.53 | 4.63 | 0.19 | <0.01 | **0.05** |
| MRT vs WSST | 1.00 | 0.89 | 8.16 | 0.26 | <0.01 | **0.05** |
| MRT vs FRT | 1.00 | 0.52 | 2.87 | 0.17 | <0.01 | **0.05** |
| SST vs WSST | 1.00 | 0.73 | 8.97 | 0.24 | <0.01 | **0.05** |
| SST vs FRT | 1.00 | 0.71 | 5.90 | 0.23 | <0.01 | **0.05** |
| WSST vs FRT | 1.00 | 0.64 | 5.66 | 0.20 | <0.01 | **0.05** |

**Table S9.** Results of differential abundance testing for bacterial phyla and diet in Australian freshwater turtles (bold values indicate significant results)

| Phylum | baseMean | log2FoldChange | lfcSE | stat | p | p.adjust |
| --- | --- | --- | --- | --- | --- | --- |
| Spirochaetota | 1.10 | -3.77 | 0.48 | -7.81 | <0.01 | **<0.01** |
| Bacteroidota | 9.24 | 0.58 | 0.11 | 5.17 | <0.01 | **<0.01** |
| Myxococcota | 0.41 | 1.46 | 0.37 | 3.96 | <0.01 | **<0.01** |
| Chlorolexota | 2.62 | -0.96 | 0.25 | -3.76 | <0.01 | **<0.01** |
| Actinobacteriota | 21.31 | -0.45 | 0.15 | -2.96 | <0.01 | **0.01** |
| Cyanobacteriota | 3.01 | 0.70 | 0.25 | 2.79 | 0.01 | **0.02** |
| Bacillota | 6.51 | 0.57 | 0.23 | 2.45 | 0.01 | **0.04** |

**Table S10.** Results of differential abundance testing for bacterial phyla and climate in Australian freshwater turtles (bold values indicate significant results)

| Pairs | Phylum | baseMean | log2FoldChange | lfcSE | stat | p | p.adjust |
| --- | --- | --- | --- | --- | --- | --- | --- |
| Hot semi-arid vs Hot-summer mediterranean | Cyanobacteriota | 2.85 | 3.01 | 0.69 | 4.39 | <0.01 | **<0.01** |
|  | Bacteroidota | 9.24 | 0.88 | 0.27 | 3.27 | <0.01 | **0.01** |
| Hot semi-arid vs Humid subtropical | Bacteroidota | 9.24 | 0.67 | 0.18 | 3.64 | <0.01 | **0.01** |
|  | Deinococcota | 8.63 | -1.12 | 0.34 | -3.25 | <0.01 | **0.01** |
| Hot semi-arid vs Oceanic | Spirochaetota | 1.10 | -4.02 | 0.97 | -4.15 | <0.01 | **<0.01** |
|  | Bacteroidota | 9.24 | 0.87 | 0.21 | 4.19 | <0.01 | **<0.01** |
|  | Actinobacteriota | 21.31 | -0.86 | 0.29 | -2.97 | <0.01 | **0.02** |
| Hot-summer mediterranean vs Humid subtropical | Cyanobacteriota | 2.85 | -3.76 | 0.59 | -6.38 | <0.01 | **<0.01** |
|  | Pseudomonadota | 48.04 | 0.82 | 0.20 | 4.17 | <0.01 | **<0.01** |
|  | Patescibacteriota | 0.99 | -1.41 | 0.48 | -2.95 | <0.01 | **0.02** |
| Hot-summer mediterranean vs Oceanic | Cyanobacteriota | 2.85 | -2.80 | 0.63 | -4.47 | <0.01 | **<0.01** |
|  | Pseudomonadota | 48.04 | 0.91 | 0.22 | 4.24 | <0.01 | **<0.01** |
|  | Actinobacteriota | 21.31 | -0.96 | 0.32 | -3.05 | <0.01 | **0.02** |
| Humid subtropical vs Oceanic | Spirochaetota | 1.10 | -3.52 | 0.59 | -5.92 | <0.01 | **<0.01** |
|  | Actinobacteriota | 21.31 | -0.63 | 0.18 | -3.43 | <0.01 | **0.01** |
|  | Cyanobacteriota | 2.85 | 0.96 | 0.29 | 3.27 | <0.01 | **0.01** |
